# Exploring the CDCA-Scd1 Axis: Molecular Mechanisms Linking the Colitis Microbiome to Neurological Deficits

**DOI:** 10.3390/ijms26052111

**Published:** 2025-02-27

**Authors:** Donglin Du, Qi Li, Zhengqiang Wei, Ziwei Wang, Lei Xu

**Affiliations:** 1 Department of Gastrointestinal Surgery, The First Affiliated Hospital of Chongqing Medical University, Chongqing 400016, China; 204748@hospital.cqmu.edu.cn (D.D.);; 2 Laboratory Animal Center, Chongqing Medical University, Chongqing 400016, China

**Keywords:** inflammatory bowel disease, colitis, gut microbiome, chenodeoxycholic acid, brain dysfunction

## Abstract

Inflammatory bowel disease is a risk factor for brain dysfunction; however, the underlying mechanisms remain largely unknown. In this study, we aimed to explore the potential molecular mechanisms through which intestinal inflammation affects brain function and to verify these mechanisms. Mice were treated with multiple cycles of 1% *w/v* dextran sulfate sodium (DSS) in drinking water to establish a chronic colitis model. Behavioral tests were conducted using the open field test (OFT), tail suspension test (TST), forced swimming test (FST), and Morris water maze test (MWM). Brain metabolomics, transcriptomics, and proteomics analyses were performed, and key target proteins were verified using qPCR and immunofluorescence. Four cycles of DSS administration induced colitis, anxiety, depression, and spatial memory impairment. The integrated multi-omics characterization of colitis revealed decreased brain chenodeoxycholic acid (CDCA) levels as well as reduced stearoyl-CoA desaturase (Scd1) gene and protein expression. Transplantation of the colitis microbiome resulted in anxiety, depression, impaired spatial memory, reduced CDCA content, decreased Scd1 gene and protein expression, and lower concentrations of monounsaturated fatty acids (MUFAs), palmitoleate (C16:1), and oleate (C18:1) in the brain. In addition, CDCA supplementation improved DSS-induced colitis, alleviated depression and spatial memory impairment, and increased Scd1 gene and protein expression as well as MUFA levels in the brain. The gut microbiome induced by colitis contributes to neurological dysfunction, possibly through the CDCA–Scd1 signaling axis. CDCA supplementation alleviates colitis and depressive behavior, likely by increasing Scd1 expression in the brain.

## 1. Introduction

Inflammatory bowel disease (IBD), encompassing Crohn’s disease and ulcerative colitis (UC), is an idiopathic, chronic, and relapsing inflammatory disorder of the intestines [1,2]. IBD affects a significant number of individuals, with nearly 1.5 million cases in North America and 2.2 million in Europe. Its annual incidence and prevalence continue to rise, posing increasing threats to both health and the overall standard of living [3,4]. Individuals with IBD not only experience abdominal pain, diarrhea, bleeding, and weight loss but also frequently contend with psychiatric comorbidities, such as depression, anxiety, and cognitive dysfunction [5,6,7]. A meta-analysis revealed that patients with IBD exhibit a prevalence rate of approximately 20% for anxiety and 15% for depression, which is higher than that in the general population [8]. Additionally, individuals with IBD display a higher incidence of cognitive impairment than age-matched individuals without IBD [9]. However, the mechanisms through which IBD leads to neurological dysfunction and psychiatric symptoms remain unclear.

Recently, an increasing number of studies have focused on the association between intestinal inflammation and brain dysfunction. Intestinal inflammation has emerged as a potential risk factor for neurodegenerative disorders, such as Parkinson’s disease (PD), Alzheimer’s disease (AD), and autism [10,11,12]. An observational study has revealed that patients with IBD exhibit changes in hippocampal neural activity, amygdala and hypothalamic volumes, and cortical stability [13,14]. Furthermore, animal studies have shown that dextran sulfate sodium (DSS)-induced colitis leads to cortical inflammation and hippocampal neurogenesis [15,16]. The term “brain–gut axis” refers to the intricate interplay between the brain and the gut, which involves nerves, hormones, the microbiome, and the immune system [17]. However, the mechanism by which intestinal inflammation connects to the brain via the brain–gut axis remains largely unclear.

The gut microbiota is recognized as a critical factor in regulating host health and contributes to the development of intestinal and extraintestinal disorders, such as neurodegenerative and neuropsychiatric diseases [18,19]. The gut microbiome produces metabolites that interact with the brain via an external secretion system [20]. IBD can lead to gut microbiome dysbiosis, which is closely associated with the pathophysiological process of intestinal inflammation [21]. This altered microbiome in IBD could lead to changes in metabolites, which may influence brain function through the bloodstream. Therefore, in this study, we used multi-omics analysis to explore the potential molecular mechanisms through which intestinal inflammation affects brain function and conducted relevant experiments to verify these mechanisms.

## 2. Results

### 2.1. DSS-Induced Colitis Lead to Gut Microbiome Alteration

**Figure 1:**
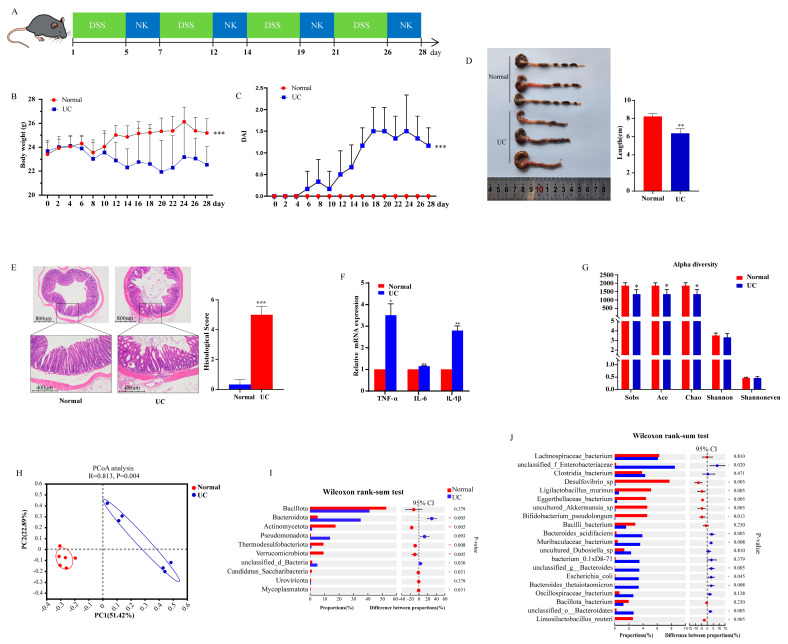
The administration of DSS induced colitis and gut microbiota alteration. (**A**) Animal model of colitis pattern diagram (NK: normal drinking); (**B**,**C**) The body weight and DAI scores between the Normal and UC groups (*n* = 6); (**D**) The colon change between the Normal and UC groups (*n* = 3); (**E**) The HE and histological scores between the Normal and UC groups (*n* = 3); (**F**) The expression of the pro-inflammatory cytokines *TNF-a*, *IL-6*, and *IL-1β* between the Normal and UC groups (*n* = 3). Data are presented as mean ± SD; *p* values were determined using Student’s *t*-test, * *p* < 0.05, ** *p* < 0.01, *** *p* < 0.001. (**G**,**H**) α-Diversity and β-diversity between the Normal and UC groups; (**I**) Significant differences in the relative abundance of the top 10 phyla; (**J**) Significant differences in the relative abundance of the top 20 species. Data are presented as mean ± SD. Alpha diversity indices (Sobs, Ace, Chao, Shannon, and Shannon evenness indices) were compared using the Wilcoxon rank-sum test. Principal coordinate analysis (PCoA) based on Bray–Curtis similarities and ANOSIM analysis were conducted to assess β-diversity. A two-tailed Wilcoxon rank-sum test with false discovery rate (FDR) and multiple comparison correction was performed at the phylum and species levels. * p < 0.05, ** p < 0.01, *** p < 0.001, *n* = 6 mice.

Firstly, we used four cycles of DSS treatment to induce the chronic colitis model (Figure 1A), where the mice in the UC group exhibited weight loss (Figure 1B), increased DAI scores (Figure 1C), and decreased colon length (Figure 1D). Histological analysis of the distal colon in the UC group revealed cellularity, disorganization, and crypt damage. The histological score of the Normal group was significantly lower than that of the UC group (Figure 1E). In addition, the levels of the pro-inflammatory cytokines *IL-6*, *IL-1β*, and *TNF-α* were significantly increased in the colonic tissues of mice from the UC group (Figure 1F). Metagenomic analysis of fecal samples revealed that the α-diversity indices of *Ace*, *Chao*, and *Sobs* were lower in the UC group than in the Normal group (Figure 1G). β-Diversity, as assessed using PCoA, revealed a different distribution of the gut microbial community between the Normal and UC groups (Figure 1H). At the phylum level, the relative abundance of *Bacteroidota* was reduced, while the relative abundances of *Actinomycetota*, *Thermodesulfobacteriota*, *Verrucomicrobiota*, *Candidatus Saccharibacteria*, and *Mycoplasmatota* were lower in the UC group than in the Normal group (Figure 1I). Furthermore, species stratification based on abundance (top 20) showed that, compared to the Normal group, the UC group had higher relative abundances of unclassified_*Enterobacteriaceae*, *Bacteroides acidifaciens*, *Muribaculaceae bacterium*, *unclassified_g_Bacteroides*, *Escherichia coli*, *Bacteroides thetaiotaomicron*, and unclassified_o_Bacteroidales and lower relative abundances of *Desulfovibrio *sp., *Ligilactobacillus murinus*, *Eggerthellaceae bacterium*, uncultured *Akkermansia *sp., *Bifidobacterium pseudolongum*, and *Limosilactobacillus reuteri* (Figure 1J).

### 2.2. DSS-Induced Colitis Leads to Neurological Dysfunction

**Figure 2:**
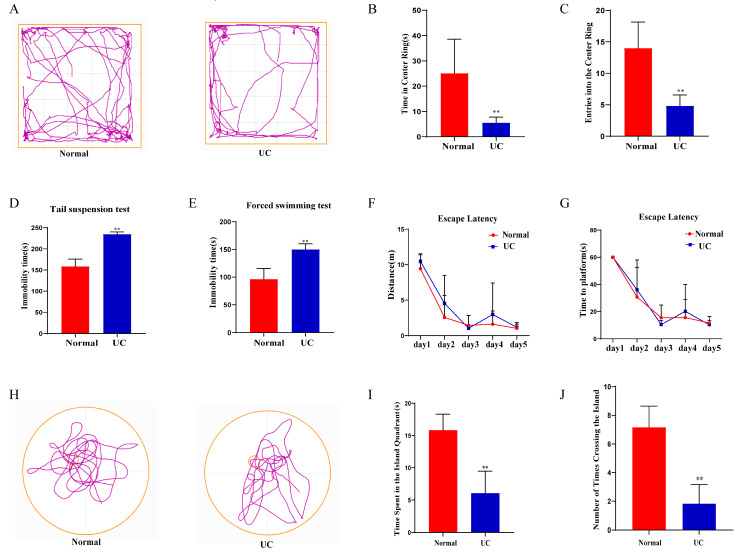
Behavioral test in mice from the Normal and UC groups. (**A**) Representative movement tracks in the open field test (The color purple symbolizes the motion trajectory, the yellow outer frame represents the periphery of OFT); (**B**,**C**) Time spent in and entries into the center of the open field between the two groups; (**D**,**E**) Immobility time in the tail suspension test (TST) and forced swimming test (FST) between the two groups; (**F**,**G**) Distance and time to find the hidden platform during the training phase of the MWM; (**H**) Movement tracks in the probe trial of the MWM (The color purple symbolizes the motion trajectory, the yellow outer circle represents the periphery of MWM, small yellow circle represents the Island); (**I**,**J**) Time spent in the target quadrant and number of times crossing the former target area in the probe trial of the MWM. Data are expressed as mean ± SD. The data were analyzed using one-way ANOVA followed by a least significant difference test (LSD) or unpaired *t*-test across groups. ** *p* < 0.01, *n* = 6 mice.

After the colitis model was successfully established, the behavioral tests were conducted. In the OFT, the time spent in and the number of entries into the center of the open field were significantly lower in the UC group compared to the Normal group (Figure 2A–C, *p* < 0.05). The immobility times in the TST and FST (Figure 2D,E, *p* < 0.05) were significantly shorter in the UC group than in the Normal group. During the training phase of the MWM, no differences were observed in the distance and time taken to find the hidden platform across training days (Figure 2F,G). However, in the probe trial for spatial memory, the time spent in the island quadrant and time taken to cross the platform island were significantly shorter in the UC group than in the Normal group (Figure 2H–J, *p* < 0.05).

### 2.3. Metabolite Changes in the Colon and Brain After Multiple-Cycle Administration of DSS

**Figure 3:**
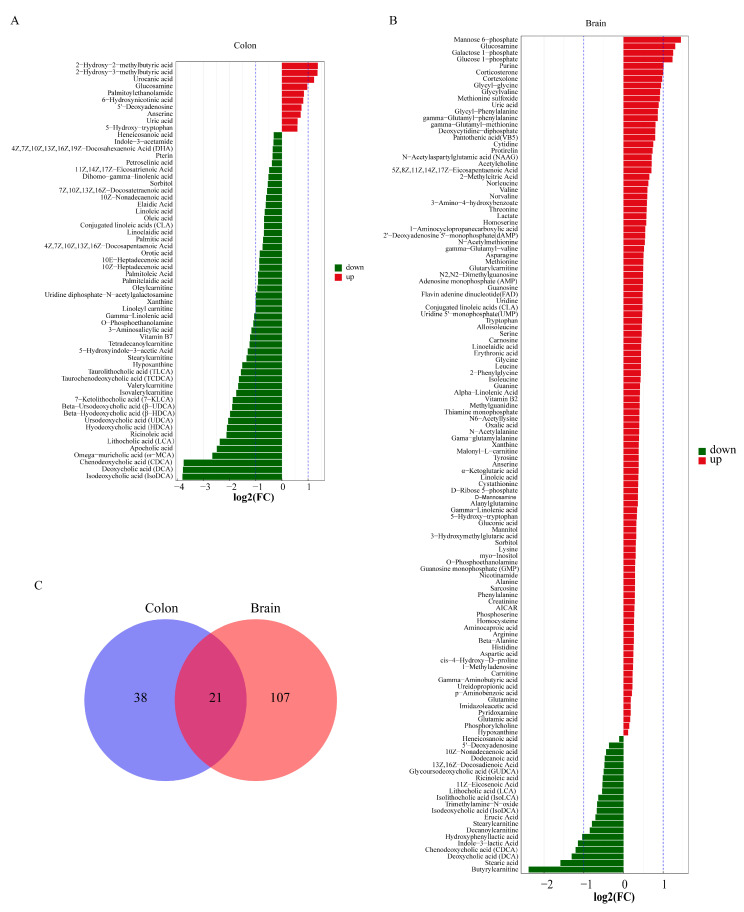
Different metabolite levels between the Normal and UC groups. (**A**,**B**) Bar graphs of different colon and brain metabolites; (**C**) Venn diagram showing overlap of colon and brain metabolites (*n* = 6 mice). Statistical significance was evaluated using an unpaired Student’s *t*-test.

Previous studies have shown that DSS-induced colitis leads to gut microbiome alterations and neurological dysfunction, indicating that the colitis-associated microbiome may induce changes in metabolites that affect the brain. Therefore, we selected the colon and brain for targeted metabolomic analysis of the microbiome to detect metabolite changes after DSS administration. To profile differential metabolism, we performed OPLS-DA and observed a clear separation between the two groups (Figure S1A,B). Permutation tests with 200 iterations showed that the models were stable. In the colon, the levels of 59 metabolites were found to differ between the two groups, among which the levels of 10 metabolites were higher and those of 49 metabolites were lower in the UC group than in the Normal group (Figure 3A). In the brain, the levels of 128 metabolites differed between the two groups, among which the levels of 21 metabolites were lower and those of 107 metabolites were higher in the UC group than in the Normal group (Figure 3B). A combined analysis of brain and colon metabolites revealed simultaneous changes in 21 metabolites (Figure 3C). Among these metabolites, lithocholic acid (LCA), isodeoxycholic acid (IsoDCA), chenodeoxycholic acid (CDCA), and deoxycholic acid (DCA), which are bile acids (BAs), were reduced simultaneously. The levels of conjugated linoleic acid, linoelaidic acid, linoleic acid, and gamma-linolenic acid, which are related to lineolic acid and its derivatives, were reduced in the colon but increased in the brain in the UC group (Table S1).

### 2.4. Transcriptome and Proteome Sequencing of the Brain After Multiple-Cycle Administration of DSS

**Figure 4:**
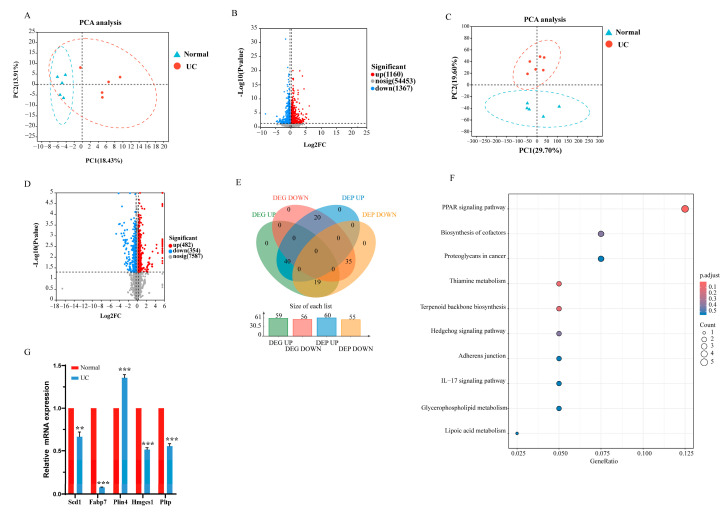
Analysis of the transcriptome and proteome sequencing of brain tissue between the Normal and UC groups. (**A**) PCA of transcriptome data between the two groups; (**B**) Volcano plot of transcriptome data between the two groups; (**C**) PCA of proteome data between the two groups. Differential expression analysis was performed using DESeq2 or DEGSeq. DEGs with |log2FC| ≥ 1.2 and FDR ≤ 0.05 (DESeq2) or FDR ≤ 0.001 (DEGseq) were considered to be significant. (**D**) Volcano plot of proteome data between the two groups; (**E**) Venn diagram showing the overlap of transcriptome and proteome data between the two groups (*n* = 5). Statistical probabilities and fold changes (FCs) for proteins between the experimental groups were calculated using the R package “*t*-test”. Differentially expressed proteins were identified based on an FC > 1.2 or < 0.83 and *p* < 0.05. (**F**) KEGG analysis of proteins and genes showing similar changes between the two groups; (**G**) qPCR analysis of PPAR signaling pathway-associated genes. Data are presented as mean ± SD, and *p* values were determined using Student’s *t*-test, ** *p* < 0.01, *** *p* < 0.001, *n* = 3 per group.

Principal component analysis (PCA) showed a clear separation of the transcriptome and proteome sequences between the two groups (Figure 4A,C). The fold difference in gene and protein expression was set to 1.2. The results revealed that the expression of 1160 genes was elevated and that the expression of 1367 genes was reduced in the UC group (Figure 4B), while the expression of 482 proteins was increased and that of 354 proteins was decreased in the UC group (Figure 4D). A joint analysis identified 115 genes that were detected in both transcriptome and proteome sequencing (Figure 4E). Of these, the expression trends of 75 proteins and genes were similar. Among these 75 proteins or genes, 40 were upregulated and 35 were downregulated in the UC group (Table S2). KEGG analysis of the 75 genes revealed major enrichment in the PPAR signaling pathway (Figure 4F), with associated genes including *Scd1*, *Fabp7*, *Plin4*, *Hmgcs1*, and *Pltp*. Additionally, the quantitative real-time PCR (qPCR) results revealed that the expression of *Scd1*, *Fabp7*, *Hmgcs1*, and *Pltp* was reduced, while the expression of *Plin4* was elevated in the UC group compared to that in the Normal group. This is consistent with the findings obtained from the transcriptome and proteome sequencing (Figure 4G).

### 2.5. Colitis Gut Microbiome Leads to Colitis and Neurological Dysfunction

**Figure 5:**
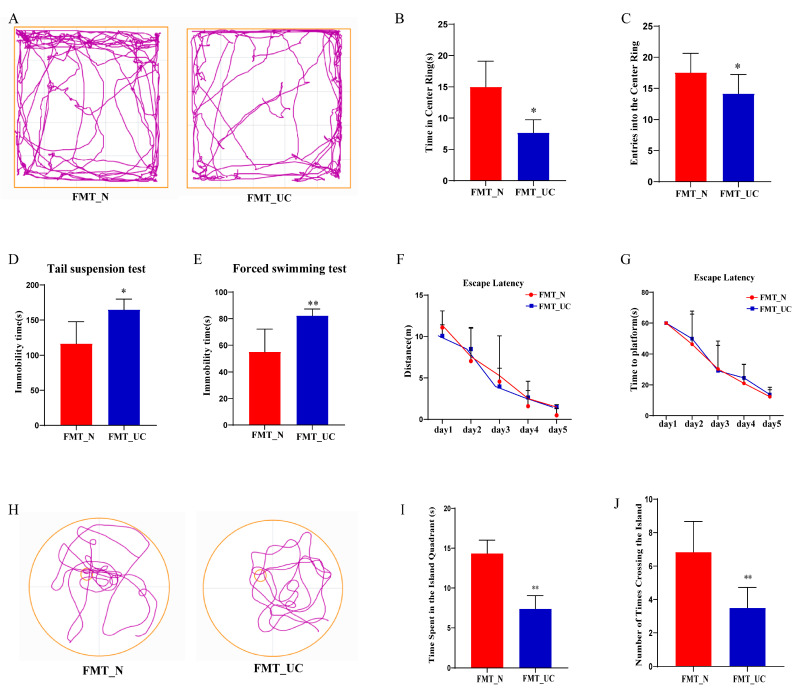
Gut microbiome induced by colitis leads to neurological dysfunction. (**A**) Representative movement tracks in the open field test between the two groups (The color purple symbolizes the motion trajectory, the yellow outer frame represents the periphery of OFT); (**B**,**C**) Time spent in and the number of entries into the center of the open field between the two groups; (**D**,**E**) Immobility time in the TST and FST tests between the two groups; (**F**,**G**) Distance and time taken to find the hidden platform during the training period of the MWM between the two groups; (**H**) Movement tracks in the probe trail of the MWM between the two groups (The color purple symbolizes the motion trajectory, the yellow outer circle represents the periphery of MWM, small yellow circle represents the Island); (**I**,**J**) Time spent in the target quadrant and number of crossings over the former target area in the probe trial of the MWM between the two groups. Each dataset is expressed as mean ± SD. The data were analyzed using one-way ANOVA followed by a least significant difference test (LSD) or unpaired *t*-test across groups. * *p* < 0.05; ** *p* < 0.01, *n* = 6 per group.

Firstly, 16s rRNA sequencing revealed that the FMT_N and FMT_UC groups had a gut microbiome similar to those of the Normal and UC groups, respectively, indicating that the gut microbiome transplantation was effective (Figure S2A–D). No significant differences were observed in weight change and colon length between the FMT_N and FMT_UC groups (Figure S2E,F). Histological analysis of the distal colon in the FMT_UC group revealed inflammatory cell infiltration (Figure S2G). Mice transplanted with the colitis microbiome showed higher expression of *IL-6*, *IL-1β*, and *TNF-α* in the colon than mice transplanted with the normal mouse microbiome (Figure S2H). The time spent in and number of entries into the center of the open field were significantly lower in the FMT_UC group than in the FMT_N group (Figure 5A–C). The immobility time in the TST and FST (Figure 5D,E) was significantly shorter in the FMT_UC group than in the FMT_N group. No differences were observed between the groups in terms of the distance and time taken to find the hidden platform during the training days (Figure 5F,G). In the probe trail, the time spent in the island quadrant and the number of crossings over the platform island were significantly lower in the FMT_UC group than in the FMT_N group (Figure 5H–J).

### 2.6. DSS-Induced Gut Microbiome Decreases the Content of CDCA and Expression of Scd1 in the Brain

**Figure 6:**
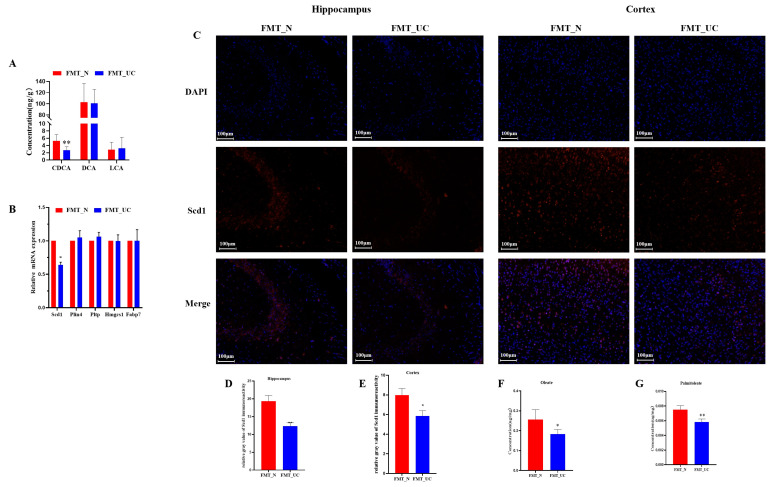
Colitis microbiome leads to colitis and decreases the expression of the *Scd1* gene and protein. (**A**) The concentrations of LCA, IsoDCA, CDCA, and DCA in the FMT_N and FMT_UC groups (*n* = 6). Statistical significance was evaluated using an unpaired Student’s *t*-test. (**B**) The relative expression of *Scd1*, *Fabp7*, *Plin4*, *Hmgcs1,* and *Pltp* between the FMT_N and FMT_UC groups. *p* values were determined using Student’s *t*-test. (**C**) Immunofluorescence images of *Scd1* in the hippocampus and cortex; (**D**,**E**) The relative gray value of *Scd1* immunoreactivity in the hippocampus (CA3) and cortex (*n* = 3). *p* values were determined using Student’s *t*-test. (**F**,**G**) Comparison of the concentration of palmitoleate (C16:1) and oleate (C18:1) in the brain between the FMT_UC and FMT_N groups (*n* = 6). Each dataset is expressed as mean ± SD. Statistical significance was evaluated using an unpaired Student’s *t*-test. * *p* < 0.05; ** *p* < 0.01.

The previous data showed that DSS-induced colitis leads to changes in the concentrations of LCA, IsoDCA, CDCA, and DCA. To assess the impact of the gut microbiome on these metabolites in the brain, we measured the LCA, IsoDCA, CDCA, and DCA levels in the FMT_N and FMT_UC groups. The results showed no apparent changes in the LCA and DCA levels between the two groups, while IsoDCA was undetectable. CDCA was significantly decreased in the FMT_UC group compared to the FMT_N group (Figure 6A), indicating that the gut microbiome is a key factor that decreased the brain CDCA content. Then, the correlation analysis of CDCA in the colon and different bacterial species (top 20) revealed that CDCA has a positive correlation with Limosilactobacillus_reuteri, Bifidobacterium_pseudolongum, and Eggerthellaceae_bacterium (Figure S3). Subsequently, we used qPCR to measure changes in brain PPAR signaling pathway–associated genes, namely, *Scd1*, *Fabp7*, *Plin4*, *Hmgcs1*, and *Pltp*. The results revealed that *Scd1* expression was significantly reduced in the FMT_UC group compared to the FMT_N group (Figure 6B). Immunofluorescence staining of the hippocampus and cortex showed that Scd1 was significantly reduced in the FMT_UC group compared to the FMT_N group (Figure 6C–E). Next, we measured the levels of palmitoleate (C16:1) and oleate (C18:1), products of Scd1, in the brain. The results showed that the C16:1 and C18:1 levels were significantly lower in the FMT_UC group than in the FMT_N group (Figure 6F,G). These data indicate that the colitis-microbiome affects the brain CDCA content and Scd1 expression, which may be associated with neurological dysfunction induced by colitis.

### 2.7. CDCA Ameliorates Colitis and Neurological Dysfunction in DSS-Induced Colitis

**Figure 7:**
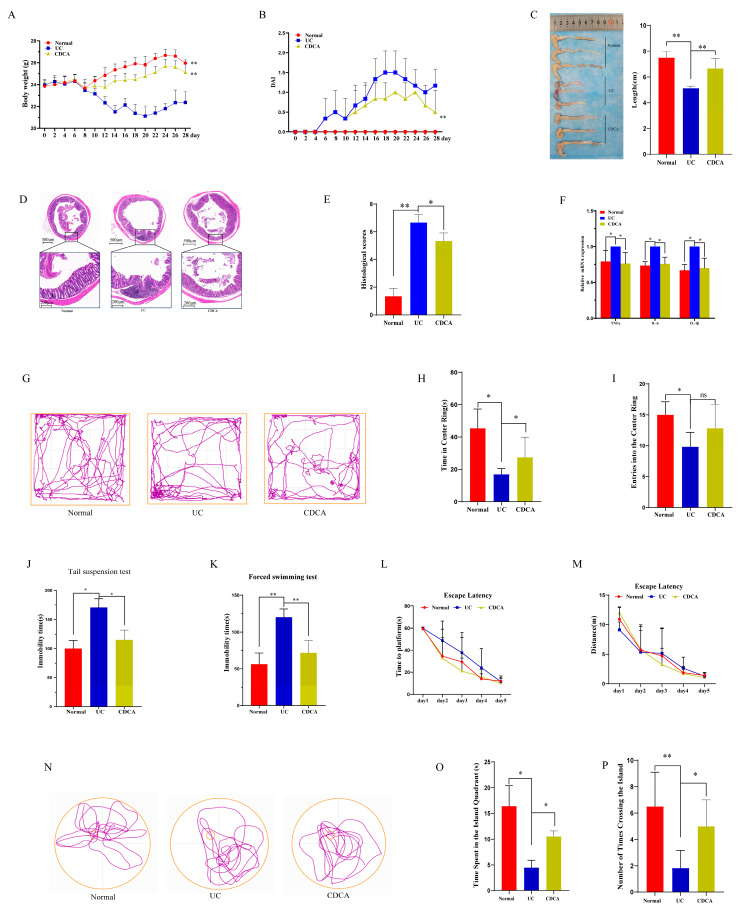
CDCA ameliorates colitis and neurological dysfunction in colitis mice. (**A**,**B**) Body weight and DAI scores among the Normal, UC, and CDCA groups (*n* = 6); (**C**) Colon length for the Normal, UC, and CDCA groups (*n* = 3); (**D**,**E**) H&E staining and histological scores for the Normal, UC, and CDCA groups (*n* = 3); (**F**) Expression of the pro-inflammatory cytokines IL-6, IL-1β, and TNF-α in the Normal, UC, and CDCA groups (*n* = 3); Data are presented as mean ± SD, and *p* values were determined using Student’s *t*-test, * *p* < 0.05, ** *p* < 0.01. (**G**) Representative movement tracks in the open field test for the Normal, UC, and CDCA groups(The color purple symbolizes the motion trajectory, the yellow outer frame represents the periphery of OFT); (**H**,**I**) Time spent in and number of entries into the center of the open field among the three groups; (**J**,**K**) Immobility times in the TST and FST among the three groups; (**L**,**M**) Distance traveled and time required to find the hidden platform during the training period of the MWM among the three groups; (**N**) Movement tracks in the probe trial of the MWM across the three groups (The color purple symbolizes the motion trajectory, the yellow outer circle represents the periphery of MWM, small yellow circle represents the Island); (**O**,**P**) Time spent in the target quadrant and the number of crossings over the former target area in the probe trial of the MWM among the three groups. Data are expressed as mean ± SD. The data were analyzed using one-way ANOVA followed by a least significant difference test (LSD) or unpaired *t*-test across groups. ns mean no significant, * *p* < 0.05, ** *p* < 0.01, *n* = 6 per group.

The above experiment indicates that colitis reduces CDCA levels in the brain and that colitis microbiome transplantation further reduces the brain CDCA levels. We examined the effects of CDCA on colitis in mice. CDCA treatment ameliorated colitis-induced weight loss, decreased DAI scores, and increased colon length (Figure 7A–C). Histological analysis showed that CDCA significantly reduced histological damage and the pro-inflammatory cytokines *IL-6*, *IL-1β*, and *TNF-α* after DSS administration (Figure 7D–F). In the OFT trials, CDCA treatment significantly increased the time spent in the center, although the number of entries into the center did not differ among the UC, CDCA, and Normal groups (Figure 7G–I). The immobility times in the TST and FST (Figure 7J,K) were significantly decreased in the CDCA group compared to the UC group. In the MWM, no differences were observed in the distance or time taken to find the platform during the first five trials (Figure 7L,M). In the probe trial, the mice in the CDCA group spent more time in the island quadrant and crossed the platform more times than the mice in the UC group (Figure 7O,P).

### 2.8. CDCA Elevates the Expression of Scd1 in the Brain of Colitis Mice

**Figure 8:**
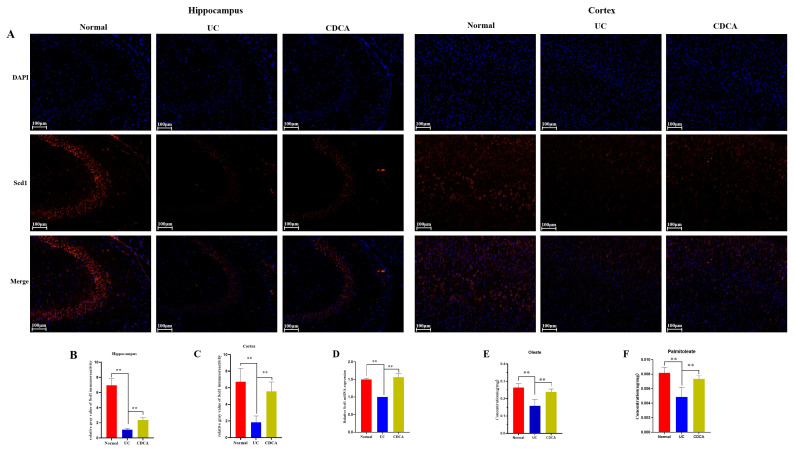
CDCA increased the expression of Scd1 in the brains of colitis mice. (**A**) Immunofluorescence images of Scd1 in the hippocampus and cortex among the Normal, UC, and CDCA groups; (**B**,**C**) The relative gray value of Scd1 immunoreactivity in the hippocampus (CA3) and cortex (*n* = 3). (**D**) Gene expression of *Scd1* in the brain among the Normal, UC, and CDCA groups (*n* = 3). Data are expressed as mean ± SD. *p* values were determined using Student’s *t*-test. ** *p* < 0.01. (**E**, **F**) Concentrations of palmitoleate (C16:1) and oleate (C18:1) in the brain among the three groups (*n* = 6). Data are expressed as mean ± SD. Statistical significance was evaluated using an unpaired Student’s *t*-test. ** *p* < 0.01.

The above experiment showed that CDCA exerts a protective effect on colitis and neurological dysfunction. Transplanting the colitis microbiome decreased the CDCA content and *Scd1* expression. We subsequently explored the effect of CDCA treatment on *Scd1* expression in the UC group. Immunofluorescence showed that Scd1 protein levels in the brain hippocampus and cortex were increased in the CDCA group compared to those in the UC group (Figure 8A–C). Gene expression of *Scd1* was also increased in the CDCA group compared to that in the UC group (Figure 8D). Palmitoleate (C16:1) and oleate (C18:1) levels were lower in the UC group than in the CDCA group (Figure 8E,F), which indirectly indicates that CDCA can regulate the expression of Scd1 in the brain.

## 3. Discussion

In this study, we investigated the impact of colitis on brain function and the role of the colitis-induced gut microbiome in neural function. First, we found that mice with colitis exhibited neurological dysfunction, and metabolomic analysis revealed a significant decrease in the CDCA content in both the colon and brain of these mice. Transcriptome and proteomic analyses revealed that the expression of Scd1 was decreased in the brains of mice with colitis. Next, we showed that transplanting the gut microbiome into antibiotic-treated mice resulted in neurological dysfunction and a decrease in the CDCA content and expression of Scd1 in the brain, suggesting a regulatory role of the gut microbiota in CDCA metabolism and Scd1 expression. Furthermore, we found that gavaging CDCA increased the expression of Scd1 in the brain and the levels of its products C16:1 and C18:1, which indicated that CDCA-SCD1 acts as an intermediate mediator in the impact of the colitis microbiome on the brain.

Patients with IBD have an increased risk of developing mood disorders, such as anxiety and depression [22]. Additionally, clinical research has revealed that patients with IBD exhibit changes in brain morphology, including gray matter volume, in parts of the frontal cortex and anterior midcingulate cortex, which are areas involved in nociception and emotional and cognitive processes [23]. These studies indicate that IBD has a clear impact on the brain. In this study, we used four-cycle administration of DSS to induce a chronic colitis mouse model, and the behavior test showed that chronic colitis mice exhibited anxiety, depressive behavior, and impaired memory function. However, chronic colitis mice did not show spatial learning loss, which is consistent with the findings of a previous study [24]. The gut microbiome plays a crucial role in brain function [25]. Our study results showed that chronic colitis mice exhibited changes in the gut microbiome, confirming that microbiome dysbiosis is a common feature of IBD [26]. Changes in the gut microbiome affect brain function, leading to psychiatric symptoms and dysfunction. In this study, we used an antibiotic cocktail to deplete the mouse microbiome while minimizing adverse impacts in terms of animal morbidity and mortality [27] and found that the mice exhibited depression, anxiety, and impaired memory after transplanting the colitis fecal microbiota, which demonstrated that the microbiome induced by colitis is a driving factor influencing brain function.

One known mechanism through which the gut communicates with the brain involves metabolites derived from gut bacteria, including short-chain fatty acids, branched-chain fatty acids, succinate, lactate, and trimethylamine N-oxide [28,29,30]. In this study, metabolomic analysis of the colon and brain showed that CDCA levels were simultaneously decreased. Additionally, CDCA levels were decreased in mice that received transplants of the colitis microbiome, suggesting that the colitis-induced microbiome is a factor impacting brain CDCA concentrations. The gut microbiome regulates the bioconversion, synthesis, and transport of BAs, affecting the composition of the BA pool [31]. Various gut bacteria, such as *Lactobacillus*, *Bifidobacterium*, *Clostridium*, and *Bacteroides*, express BA-related genes, including bile salt hydrolase (BSH) and 7α-dehydroxylase enzyme, which can regulate BA metabolism processes [32]. Our study results revealed that colitis causes a decrease in Limosilactobacillus_reuteri and Bifidobacterium_pseudolongum, which have a BSH enzyme to remove glycine (Gly) and taurine (Tau) from conjugated bile acids to produce free CDCA, which may be a reason for the decreased CDCA levels in the colon and brain. Moreover, patients with AD have been found to have an altered BA profile, which is closely associated with cognition decline [33]. BAs are present in the brain, and evidence indicates that they cross the blood–brain barrier, causing physiological changes [34]. Some BAs, such as ursodeoxycholic acid, exert beneficial effects [35]. Our study demonstrated that supplementation with CDCA also ameliorated neural function in colitis mice. CDCA is thought to improve AD neurotoxicity and cognitive decline by enhancing insulin signaling [36],which shows that CDCA exerts beneficial effects on the brain.

Transcriptome and proteome sequencing of mouse brains showed that the expression of Scd1 was significantly lower in mice with colitis. A previous study showed that Scd1 gene expression is decreased in the brains of aging rats, implying that *Scd1* is related to cognition [37]. Scd1 is a rate-limiting enzyme that catalyzes the biosynthesis of MUFAs, including palmitoleate (C16:1) and oleate (C18:1) [38]. Our study showed that the concentration of MUFAs in the brains of mice with colitis decreased, and the concentration of MUFAs was also reduced after transplanting the gut microbiome, which contributed to the reduction in MUFAs in colitis. MUFAs exert beneficial effects on cortical activity, locomotion, and sleep [39]. A study showed that supplementation with MUFAs extends lifespan, indicating that MUFAs exert beneficial effects on the brain [40]. Our study showed that transplanting the colitis mouse microbiome results in a decrease in Scd1 expression and MUFAs, and supplementation with CDCA improved the expression of Scd1 and the concentration of MUFAs in the brains of mice with colitis. This indicates that the colitis microbiome impacts brain function, possibly through the CDCA–Scd1 signaling pathway; however, further experiments are needed to verify this. In addition, further studies are required to identify whether the human colitis microbiome can cause a decrease in the CDCA content and Scd1 protein expression.

This study has some limitations. First, the mouse model, while a useful tool, has inherent limitations in fully replicating the complexity of human disease. Future studies involving human participants are essential to validate these findings. Second, our study focused on a specific set of metabolites and signaling pathways, and a broader metabolomic and transcriptomic analysis could reveal additional mechanisms. Third, the short-term study design may not fully capture the long-term consequences of gut microbiota alterations on neurological function. Therefore, long-term studies are needed to assess chronic effects and to investigate potential therapeutic interventions. Fourth, future research should further explore whether CDCA rescues the metabolic disturbances caused by colitis and clarify whether its therapeutic effects are driven by the modulation of bile acid metabolism or downstream targets involved in neurological function. Fifth, given the animal welfare, a low number of animals were utilized in each experiment.

## 4. Materials and Methods

### 4.1. Experimental Animal Model of Colitis and Treatment

Male C57BL/6J mice (6 weeks) were acquired from the Laboratory Animal Center of Chongqing Medical University (Chongqing, China) and housed under specific pathogen-free conditions with cyclical 12 h light–dark intervals at 25 °C. Chronic colitis was induced in the mice according to the procedure described previously [41]. The mice were divided into the Normal and UC groups (*n* = 6 per group). Mice in the UC group were administered four cycles of 1% (wt) DSS (MPbio, Santa Ana, CA, USA)(molecular weight: 30,000–50,000 g/mol) in drinking water for the initial 5 days, followed by regular drinking water for the subsequent 2 days. The mice were divided into the Normal, UC, and chenodeoxycholic acid (CDCA) groups (*n* = 6 per group). The UC and CDCA groups were subjected to chronic colitis model induction as previously described. Additionally, CDCA (Yuanye Bio-technology Co., Ltd., Shanghai, China) was dissolved in saline and diluted to 50 mg/kg for oral gavage during the duration of the CDCA group model establishment [42]. Alterations in the disease activity index (DAI) were measured as previously described (*n* = 6 per group) [43]. Distal colon samples were fixed in 10% formaldehyde and subjected to staining using hematoxylin and eosin (*n* = 3 per group). Colon histology scores were classified into four categories based on a prior study: diminution of epithelial integrity, cryptic structural compromise, depletion of mucus-secreting cells, and leukocyte infiltration (*n* = 3 per group) [44].

### 4.2. Behavioral Tests (Stoelting, Kiel, WI, USA)

#### 4.2.1. Morris Water Maze Task

The water maze task (MWM) is a test used to assess the effects of spatial learning and memory and was performed as previously described (*n* = 6 per group) [45]. Briefly, the mice underwent four trials (up to 60 s) during five non-stop training days, followed by a single 60 s probe trial on day 6. The latency to locate the platform during the training days, the number of traversals over the target area, and the duration spent in the target quadrant during the probe trial were recorded.

#### 4.2.2. Open Field Test

Open field tests (OFTs) were used to measure anxiety behavior in rodents and were conducted as previously described (*n* = 6 per group) [45]. The experimental setup consisted of a square arena measuring 50 × 50 cm, enclosed by 40 cm high walls. An overhead video camera was employed to capture the spontaneous motor activity of the mice during a 5 min trial. Mice were positioned at the center of the arena, allowing for the tracking of the time spent in the center and the frequency of entries into the center.

#### 4.2.3. Tail Suspension Test

In the tail suspension test (TST), which is a measurement of depressive behavior, each mouse was individually suspended via its tail 2 cm from the tip, ensuring its head was positioned more than 10 cm above the apparatus floor. After a 2 min adaptation phase, the immobility time was documented over a 4 min period using a video tracking system (*n* = 6 per group) [46].

#### 4.2.4. Forced Swimming Test

In the forced swimming test (FST), a test to assess depressive behavior, mice were individually positioned in a transparent acrylic tank (height: 40 cm, diameter: 30 cm) containing water. The water depth was 20 cm, and the temperature was maintained at 23 ± 2 °C. The FST lasted for 6 min, with the first 2 min allocated for adaptation. The immobility duration during the final 4 min was recorded using a video tracking system (*n* = 6 per group) [47].

### 4.3. Antibiotic Treatment Protocol and Fecal Microbiota Transplant Protocol

The mice were administered an antibiotic mixture comprising ampicillin (1 g/L) (Topscience, Shanghai, China), vancomycin (0.5 g/L) (Topscience, Shanghai, China), neomycin (1 g/L) (Topscience, Shanghai, China), and metronidazole (1 g/L) (Topscience, Shanghai, China) in drinking water supplemented with 10% sucrose for 21 days before fecal microbiota transplanting (FMT) [48]. Fecal microbiota preparations were derived from fresh mouse stool samples. In brief, fecal samples obtained from both Normal mice and mice subjected to four cycles of DSS treatment were dissolved in sterile saline at a concentration of 100 mg/mL and mixed thoroughly by vortexing. Subsequently, the suspension was filtered through a 70 µm membrane and centrifuged at 800× *g* for 3 min (repeated twice). The supernatant was then collected and administered to the antibiotic-treated mice via oral gavage for a period of 19 days (100 µL per mouse) [49]. The Normal and DSS-treated mice, which were used to provide the donor fecal microbiota, were defined as the D_N and D_UC groups (*n* = 6 per group). Mice with transplanted normal and colitis mouse gut microbiomes were defined as the FMT_N and FMT_UC groups, respectively (*n* = 6 per group).

### 4.4. Shotgun Metagenomic and 16S rRNA Sequencing

#### 4.4.1. Shotgun Metagenomic Sequencing

Fecal samples from the Normal and UC groups were collected 29 days after four cycles of DSS treatment (*n* = 6 per group). The collected samples were promptly transferred into sterile tubes and kept on ice prior to storage at −80 °C. The subsequent processes—including fecal genomic DNA extraction, polymerase chain reaction (PCR) amplification, and sequencing—were conducted by Majorbio BioPharm Technology Co., Ltd. (Shanghai, China) [50]. Detailed protocols are available in the Supplementary Material. Data were analyzed using the free online Majorbio Cloud Platform (www.majorbio.com) (accessed on 10 November 2023), and the associated metagenomic sequencing data have been archived in the NCBI Short Read Archive database (SUB14893322).

#### 4.4.2. Fecal 16s rRNA Sequencing

Fecal genomic DNA extraction, PCR amplification, and sequencing were performed by Majorbio BioPharm Technology Co., Ltd. (Shanghai, China) [50], and the detailed steps are described in the Supplementary Material. Raw sequencing reads were deposited in the NCBI Sequence Read Archive database (SUB14891620). The purified amplicons were combined in equimolar ratios and subjected to paired-end sequencing on an Illumina NextSeq 2000 PE300 platform (Illumina, San Diego, CA, USA) following standardized protocols established by Majorbio BioPharm Technology Co., Ltd.

#### 4.4.3. Data Analysis

Alpha diversity indices (*Sobs*, *Ace*, *Chao*, *Shannon*, and *Shannon evenness* indices) were compared using the Wilcoxon rank-sum test. Principal coordinate analysis (PCoA) based on Bray–Curtis similarities and ANOSIM analysis were conducted to assess β-diversity. A two-tailed Wilcoxon rank-sum test with false discovery rate (FDR) and multiple comparison correction was performed at the phylum and species levels.

### 4.5. Metabonomic Analysis Based on LC/MS

Colon and brain samples from the Normal and UC groups were collected on day 29 following four cycles of DSS treatment (*n* = 6 per group). The samples were rapidly cryopreserved in liquid nitrogen following dissection. The tissues were cut on dry ice (~50 mg) and placed into 2 mL Eppendorf tubes. Metabolite extractions and liquid chromatography with tandem mass spectrometry (LC/MS) analysis were conducted by Shanghai Applied Protein Technology Inc. [51]. The detailed procedure is described in the Supplementary Material. The quantitative data were processed using the MultiQuant or Analyst program. Quality controls were analyzed concurrently with the biological specimens. Metabolites in quality control specimens with coefficients of variation below 30% were categorized as reproducible measurements. Following sum normalization, the processed dataset was imported to SIMCA-P (version 14.1, Umetrics, Umea, Sweden) for multidimensional data examination, including orthogonal partial least-squares discriminant analysis (OPLS-DA). The model’s validity was analyzed through septuple cross-validation and response permutation testing. Variable importance in projection (VIP) scores for each parameter in the OPLS-DA model were determined to quantify their impact on classification. Statistical significance was evaluated using an unpaired Student’s *t*-test.

### 4.6. Transcriptome

Brain samples from the Normal and UC groups were collected on day 29 (*n* = 5 per group). The specimens were promptly frozen in liquid nitrogen following dissection and preserved at −80 °C. RNA extraction, library preparation, and sequencing were performed by Majorbio BioPharm Technology Company. The procedures are detailed in the Supplementary Material. Raw paired-end reads were trimmed, and quality control was executed using Fastp with default settings. Clean reads were aligned to the reference genome in orientation mode using HISAT2 software (http://ccb.jhu.edu/software/hisat2/index.shtml) (accessed on 12 November 2023). Mapped reads for each specimen were assembled using StringTie with a reference-based methodology. To ascertain differentially expressed genes (DEGs) between two groups, we determined the expression level of every transcript using the transcripts per million method. RSEM was used to quantify gene abundances. Differential expression analysis was performed using DESeq2 or DEGSeq. DEGs with |log2FC| ≥ 1.2 and FDR ≤ 0.05 (DESeq2) or FDR ≤ 0.001 (DEGseq) were considered to be significant.

### 4.7. Proteomics Analysis Based on Data-Independent Acquisition (DIA) Mass Detection

Brain samples from the Normal and UC groups were collected on day 29 after four cycles of DSS treatment (*n* = 5 per group). Protein extraction, digestion, peptide desalting and quantification, and DIA mass detection were conducted by Majorbio BioPharm Technology Co., Ltd. (Shanghai, China). The detailed procedure for sequencing is provided in the Supplementary Material. Spectronaut software (version 18) was used to search raw DIA data. For quantitative analysis, six peptides per protein and three fragment ions per peptide were selected. The parameters were as follows: protein FDR < 0.01, peptide FDR ≤ 0.01, peptide confidence ≥ 99%, and extracted ion chromatogram width ≤ 75 ppm. Shared and modified peptides were excluded, and peak areas were calculated and aggregated to obtain quantitative data. Protein identification was limited to those with at least one distinctive peptide. Proteomic data underwent bioinformatic analysis using the Majorbio Cloud Platform (https://cloud.majorbio.com) (accessed on 12 November 2023). Statistical probabilities and fold changes (FCs) for proteins between the experimental groups were calculated using the R package “*t*-test.” Differentially expressed proteins were identified based on an FC > 1.2 or < 0.83 and *p* < 0.05.

### 4.8. GC-MS Analysis of Monounsaturated Fatty Acids

A gas chromatography–mass spectrometry (GC-MS) system (Agilent Technologies, Santa Clara, CA, USA) was used to analyze the sample spectrum with the default parameters for monounsaturated fatty acid (MUFA) integration (*n* = 6 per group). The procedure for fatty acid extraction and GC-MS analysis is detailed in the Supplementary Material. Masshunter software (v10.0.707.0; Agilent, USA) was used to identify and quantify analytes in the GC-MS system following quality control protocols. A linear regression standard curve for determining specimen concentrations was created by plotting the peak area of the analyte’s mass spectrum on the *y*-axis and the analyte concentration on the *x*-axis. The peak areas of the analytes in the mass spectrum of the specimens were inserted into the linear equation to determine their concentrations.

### 4.9. Immunofluorescence Staining

Mice (*n* = 3 per group) were thoroughly anesthetized and perfused with phosphate-buffered saline followed by 4% paraformaldehyde for perfusion fixation. Subsequently, excised encephalic tissues underwent secondary fixation in 4% (*w*/*v*) paraformaldehyde for 72 h, followed by embedding in an optimal cutting temperature medium for cryosectioning. Next, 20 μm thick coronal sections were prepared and subjected to immunofluorescence labeling using established indirect methodologies as previously described [52]. The primary antibody employed was mouse anti-glial stearoyl-CoA desaturase 1 (*Scd1*) at a dilution of 1:500 (Proteintech, China). Staining intensity was quantified using Image J software (https://imagej.net/ij/).

### 4.10. Quantitative Real-Time PCR

The total RNA was isolated from mice (*n* = 3 per group) utilizing TRIzol reagent (Invitrogen, Carlsbad, CA, USA) and quantified via spectrophotometry. Complementary DNA was generated using the cDNA Synthesis SuperMix (Biotool, Houston, TX, USA) following the manufacturer’s protocol. Quantitative reverse transcription PCR was conducted using a real-time PCR system (ABI 7500, Thermo Fisher Scientific, Waltham, MA, USA) incorporating a fluorescent marker (SYBR Green; Biotool, Houston, TX, USA). The cycle threshold (Ct) score was standardized against that of glyceraldehyde 3-phosphate dehydrogenase (GAPDH) within the identical specimen. Gene expression was quantified employing the comparative CT (2^−ΔΔCT^) method. The forward and reverse primer sequences for each gene (*IL-6*, *IL-1β*, *TNF-α*, *Scd1*, *Fabp7*, *Plin4*, *Hmgcs1*, and *Pltp*) were sequenced by Sangon Biotech (Shanghai, China). The primer sequences (5′–3′) used were as follows:

GAPDH (AGGTCGGTGTGAACGGATTTG and GGGGTCGTTGATGGCAACA);
IL-6 (CCAAGAGGTGAGTGCTTCCC and CTGTTGTTCAGACTCTCTCCCT);
IL-1β (GCAACTGTTCCTGAACTCAACT and ATCTTTTGGGGTCCGTCAACT);
TNF-α (GACGTGGAACTGGCAGAAGAG and TTGGTGGTTTGTGAGTGTGAG);
Scd1 (TTCTTGCGATACACTCTGGTGC and CGGGATTGAATGTTCTTGTCGT);
Fabp7 (GGACACAATGCACATTCAAGAAC and CCGAACCACAGACTTACAGTTT);
Plin4 (GTGTCCACCAACTCACAGATG and GGACCATTCCTTTTGCAGCAT);
Hmgcs1 (AACTGGTGCAGAAATCTCTAGC and GGTTGAATAGCTCAGAACTAGCC);
Pltp (CTTCCCTCTGAAGGAGGACAA and GGAAAAGGCCACGTACACCAT).

### 4.11. Statistical Analysis

All data are presented as means ± standard deviations and were analyzed using IBM SPSS, version 21, software (IBM Corp., Armonk, NY, USA). Data from the behavioral test were analyzed using one-way ANOVA followed by a least significant difference test (LSD) or unpaired *t*-test across groups. Statistical significance was set at *p* < 0.05. All statistical figures were generated using GraphPad Prism 8.0 software (GraphPad Software, Inc., San Diego, CA, USA).

## 5. Conclusions

In conclusion, our study revealed that DSS-induced colitis leads to neurological dysfunction and decreased CDCA and Scd1 expression. Oral gavage of CDCA ameliorates colitis and neurological dysfunction and increases MUFAs by elevating Scd1 expression in the brain. In addition, transplantation of the colitis microbiome causes neurological dysfunction and a decrease in CDCA, as well as the expression of Scd1 and MUFAs in the brain. However, further studies are required to determine whether the colitis microbiome affects brain function through the microbiome–CDCA–Scd1 signaling pathway.

## Data Availability

The data underlying this article will be shared upon reasonable request to the corresponding author.

## Supplementary Materials

The following supporting information can be downloaded at: https://www.mdpi.com/article/10.3390/ijms26052111/s1.

## Abbreviations

DSS	Dextran Sulfate Sodium
CDCA	Chenodeoxycholic Acid
Scd1	Stearoyl-CoA desaturase
MUFA	Monounsaturated fatty acid
IBD	Inflammatory bowel disease
UC	Ulcerative colitis
DCA	Deoxycholic acid
DAI	Disease Activity Index
MWM	Water maze task
OFT	Open field test
TST	Tail Suspension Test
FST	Forced Swimming Test
FMT	Fecal Microbiota Transplanting
FC	Fold change
NK	Normal Drinking
OPLS-DA	Orthogonal Partial Least-Squares Discriminant Analysis
PD	Parkinson’s Disease
AD	Alzheimer’s Disease
BA	Bile acid
