# Supplementary material for: Exploring the CDCA-Scd1 Axis: Molecular Mechanisms Linking the Colitis Microbiome to Neurological Deficits
Source: Int J Mol Sci. 2025 Feb 27;26(5):2111. doi: 10.3390/ijms26052111 (PMC11900004; doi:10.3390/ijms26052111)
Supplement: Supplementary file 1 [file ijms-26-02111-s001.zip › ijms-3465989-supplementary.pdf]

1.Materials and methods

1.1 Shotgun metagenomic sequencing

1.1.1 DNA extraction and Metagenomic sequencing

0.2g of stool material was used to extract total genomic DNA with the FastPure Stool DNA Isolation Kit（Magnetic bead） (MJYH, shanghai, China) according to manufacturer′ sinstructions. Concentration and purity of extracted DNA was determined with SynergyHTX and NanoDrop2000, respectively. DNA quality was checked on 1% agarosegel.Then DNA extract was fragmented to an average size of about 350 bp using Covaris M220 (Gene Company Limited, China) for paired-end library construction. Paired-end library was constructed using NEXTFLEX Rapid DNA-Seq (Bioo Scientific, Austin, TX, USA). Paired-end sequencing was performed on Illumina NovaSeq™ X Plus (Illumina Inc., San Diego, CA, USA) at Majorbio Bio-Pharm Technology Co., Ltd. (Shanghai, China) using NovaSeq X Series 25B Reagent Kit according to the manufacturer ′ s instructions (www.illumina.com).

1.1.2 Processing of Sequencing Data.

The raw sequencing reads were trimmed of adapters, and low-quality reads (length<50 bp or with average quality value <20) were removed by fastp (https://github.com/OpenGene/fastp, version 0.20.0). Reads were aligned to the mice genome by BWA (https://mart.ensembl.org/Mus_musculus/Info/Index) and any hit associated with the reads and their mated reads were removed. The quality-filtered data were assembled using MEGAHIT (https://github.com/voutcn/megahit, version 1.1.2). Contigs with a length ≥ 300 bp were selected as the final assembling result. Open reading frames (ORFs) from each assembled contigs were predicted using Prodigal （https://github.com/hyattpd/Prodigal，version2.6.3） and a length ≥ 100 bp ORFs were retrieved. A non-redundant gene catalog was constructed using CD-HIT (http://weizhongli-lab.org/cd-hit/,version 4.7) with 90% sequence identity and 90% coverage. Gene abundance for a certain sample was eatimated by SOA Paligner (https://github.com/ShujiaHuang/SOAPaligner , version soap2.21release) with 95%identity.

1.2 16srRNA

1.2.1 DNA extraction and PCR amplification

Total microbial genomic DNA was extracted from mice samples using the FastPure Stool DNA Isolation Kit (MJYH, shanghai, China) according to manufacturer's instructions. The quality and concentration of DNA were determined by 1.0% agarose gel electrophoresis and a NanoDrop® ND-2000 spectrophotometer (Thermo Scientific Inc., USA) and kept at -80 ℃ prior to further use.. The hypervariable region V3-V4 of the bacterial 16S rRNA gene were amplified with primer pairs 338F (5'-ACTCCTACGGGAGGCAGCAG-3') and 806R(5'-GGACTACHVGGGTWTCTAAT-3') by an T100 Thermal Cycler (BIO-RAD, USA). The PCR reaction mixture including 4 μL 5 × Fast Pfu buffer, 2 μL 2.5 mM dNTPs, 0.8 μL each primer (5 μM), 0.4 μL Fast Pfu polymerase, 10 ng of template DNA, and ddH2O to a final volume of 20 μL. PCR amplification cycling conditions were as follows: initial denaturation at 95 ℃ for 3 min, followed by 27 cycles of denaturing at 95 ℃ for 30 s, annealing at 55 ℃ for 30 s and extension at 72 ℃for 45 s, and single extension at 72 ℃ for 10 min, and end at 4 ℃. All samples were amplified in triplicate. The PCR product was extracted from 2% agarose gel and purified. Then quantified using Synergy HTX (Biotek, USA).

1.2.2 Illumina sequencing and Amplicon sequence processing and analysis

Purified amplicons were pooled in equimolar amounts and paired-end sequenced on an Illumina NextSeq 2000 PE300 platform (Illumina, San Diego,USA).After demultiplexing, the resulting sequences were quality filtered with fastp (v0.19.6) and merged with FLASH (v1.2.11) . Then the high-quality sequences were denoised using DADA2 plugin in the Qiime2 (version 2020.2) pipeline with recommended parameters, which obtains single nucleotide resolution based on error profiles within samples. DADA2) denoised sequences are usually called amplicon sequence variants (ASVs). To minimize the effects of sequencing depth on alpha and beta diversity measure, the number of sequence from each sample was rarefied to 20,000, which still yielded an average Good's coverage of 97.90%. Taxonomic assignment of ASVs was performed using the Naive bayesconsensus taxonomy classifier implemented in Qiime2 and the SILVA 16S rRNA database (v138). The metagenomic function was predicted by PICRUSt2 (Phylogenetic Investigation of Communities by Reconstruction of Unobserved States) based on ASV representative sequences. PICRUSt2 is a software containing a series of tools as follows: HMMER was used to aligns ASV representative sequences with reference sequences. EPA-NG and Gappa were used to put ASV representative sequences into a reference tree. The castor was used to normalize the 16S gene copies. MinPath was used to predict gene family profiles, and locate into the gene pathways.

1.3 Targeted Metabolomics

1.3.1 Metabolite Extractions

To extract metabolites from tissue samples, 1000 μL of cold Methanol / acetonitrile / water (2:2:1, v/v) extraction solvent was added to 80 mg sample, and adequately vortexed. For absolute quantification of the metabolites, stock solutions of stable-isotope internal standards were added to the extraction solvent simultaneously. The samples was homogenized by MP homogenizer (24×2, 6.0 M/S, 60 s, twice) and sonicated at 4°C (30min/once, twice) then centrifuged at 14,000 g for 20 minutes at 4°C and the supernatant was dried in a vacuum centrifuge at 4°C. For LC-MS analysis, the samples were redissolved in 100 μL acetonitrile/water (1:1, v/v) solvent and centrifuged at 14000 g at 4 ℃ for 15 min, then the supernatant was injected.

1.3.2 LC-MS/MS Analysis

Analyses were performed using an UHPLC (1290 Infinity LC, Agilent Technologies) coupled to a QTRAP MS (6500+, Sciex) in Shanghai Applied Protein Technology Co., Ltd. The analytes were separated on HILIC (Waters UPLC BEH Amide column, 2.1 mm × 100 mm, 1.7μm) and C18 columns (Waters UPLC BEH C18-2.1x100 mm, 1.7 μm). For HILIC separation, the column temperature was set at 35 ℃; and the injection volume was 2 μL. Mobile phase A: 90% H2O + 2 mM ammonium formate + 10% acetonitrile , mobile phase B: 0.4% formic acid in acetonitrile. A gradient (85% B at 0-1 min, 80% B at 3-4 min, 70% B at 6 min, 50% B at 10-15.5 min, 85% B at 15.6 -23 min) was then initiated at a flow rate of 300 μL/min. For RPLC separation, the column temperature was set at 40℃, and the injection volume was 2 μL. Mobile phase A: 5 mM ammonium acetate in water, mobile phase B: 99.5% acetonitrile.A gradient (5% B at 0 min, 60% B at 5 min, 100% B at 11-13 min, 5% B at 13.1-16 min) was then initiated at a flow rate of 400 μL/min. The sample was placed at 4 ℃ during the whole analysis process. 6500+ QTRAP

(AB SCIEX) was performed in positive and negative switch mode. The ESI positive source conditions were as follows: Source temperature: 580°C; Ion Source Gas1 (GS1): 45; Ion Source Gas2 (GS2): 60; Curtain Gas (CUR): 35; IonSpray Voltage（IS）: +4500 V； The ESI negative source conditions were as follows: Source temperature: 580℃; Ion Source Gas1 (GS1): 45; Ion Source Gas2 (GS2): 60; Curtain gas (CUR): 35; IonSpray Voltage（IS）: -4500 V. MRM method was used for mass spectrometry quantitative data acquisition. The MRM ion pairs are showed in the attached file. A polled quality control (QC) samples were set in the sample queue to evaluate the stability and repeatability of the system.

1.4 Transcriptome

1.4.1 RNA extraction and Library preparation and Sequencing

Total RNA was extracted from the tissue using TRIzol® Reagent according the manufacturer' s instructions. Then RNA quality was determined by 53 0 0 Bioanalyser(Agilent) and quantified using the ND-2 0 0 0 (NanoDrop Technologies). Only high-quality RNA sample(OD260/280= 1.8~2.2, OD260/230≥2.0, RQN≥ 6.5, 28S:18S≥ 1.0, >1g) was used to construct sequencing library.The RNA-seq transcriptome librariy was prepared following Illumina® Stranded mRNA Prep Ligation from Illumina (San Diego, CA) using 1μg of total RNA. Shortly, messenger RNA was isolated according to polyA selection method by oligo(dT) beads and then fragmented by fragmentation buffer firstly. Secondly double- stranded cDNA was synthesized using a SuperScript double-stranded cDNA synthesis kit (Invitrogen, CA) with random hexamer primers (Illumina). Then the synthesized cDNA was subjected to end-repair, phosphorylation and 'A' base addition according to Illumina's library construction protocol. Libraries were size selected for cDNA target fragments of 300 bp on 2% Low Range Ultra Agarose followed by PCR amplified using Phusion DNA polymerase (NEB) for 15 PCR cycles. After quantified by Qubit 4.0, paired-end RNA-seq sequencing library was sequenced with the NovaSeq X plus sequencer (2 × 150bp read length).

1.5 Proteomics

1.5.1 Total protein extraction

Take out the samples in the frozen state and put it on ice. The samples were suspended in protein lysis buffer (8M urea,1% SDS) which included appropriate protease inhibitor to inhibit protease activity and the mixture were treated by high-flux tissue grinding machine for 3 times, 40 s each. Then the mixture was incubated on ice for 30 min, during which was vortex mixed for 5-10 severy 5 min. After centrifugation at 16000g at 4°C for 30min, the concentration of protein from the supernatant collected was determined by Bicinchoninic acid (BCA) method by BCA Protein Assay Kit（Thermo Scientific). Protein quantification was performed according to the kit protocol. After protein quantification, SDS-PAGE electrophoresis was performed.

1.5.2 Protein digestion

100 μg protein re-suspended with Triethylammonium bicarbonate buffer(TEAB) which with the final concentration of 100mM. The mixture was reduced with Tris(2-carboxyethyl)phosphine (TCEP) which with the final concentration of 10mM at 37 °C for 60min and alkylated with iodoacetamide (IAM) which with the final concentration of 40mM at room temperature for 40min in darkness. After centrifugation at 10000g at 4°C for 20min, the pellet

was collected, which re-suspended with 100 μL Triethylammonium bicarbonate buffer(TEAB) which with the final concentration of 100mM. Trypsin was added at 1:50 trypsin-to-protein mass ratio and incubated at 37 °C overnight.

1.5.3 Peptide desalting and quantification

After trypsin digestion, the peptides were dried by vacuum pump. Then, the enzymatically drained peptides were re-solubilized with 0.1% trifluoroacetic acid (TFA), and the peptides were desalted with HLB and dried by vacuum concentrator. Finally, the peptides were quantified using the NANO DROP ONE(Thermo Scientific) by UV absorption value .

1.5.4 DIA mass detection

Based on peptide quantification results, the peptides were analyzed by an VanquishNeo coupled with an Orbitrap Astral mass spectrometer (Thermo, USA) at Majorbio Bio-Pharm Technology Co. Ltd.(Shanghai, China). Briefly, the uPAC High Throughptu column (75 μm×5.5 cm, Thermo, USA) was used with solvent A (water with 2% ACN and 0.1% formic acid) and solvent B (water with 80% ACN and 0.1% formic acid). The peptides were eluted using the 180 SPD.Data-independent acquisition (DIA) data were acquired using an Orbitrap Astral mass spectrometer operated in DIA mode. The detection was carried out over a mass range of 70-1050 m/z (MS1), and 150-2000 m/z (MS2).

1.6 GC-MC analysis of monounsaturated fatty acids (MUFAs)

100 mg brain sample was accurately weighed and placed in a 2 mL grinding tube. A small steel ball and 1 mL of dichloromethane:methanol (v/v=1:1) both were added to the tube. Then, the tube was placed in a cryogenic grinding machine for grinding at 50 Hz for 3 min, ultrasonicated at low temperature for 15 min and standed at -20 oC for 15 min, finally centrifuged at 13000 rfc, 4 ℃ for 10 min. The supernatant was absorbed 500 μL into 1.5 mL EP tube and dried with nitrogen. After that, 0.5 mL sodium hydroxide methanol solution (0.5 mol/L) was added into the tube, vortexed for 30 s and incubated at 60 oC for 30 min. After cooling, 0.5 mL hexane was added into the tube and then vortexed for 30 s, centrifuge at 13000 rcf at 4 ℃ for 10 min. 100 μL of the upper layer solution (hexane layer) was put into a sample bottle for GC-MS analysis.Then the sample were separated with a Agilent DB-FastFAME (20 m×0.18 mm×0.20 μm) capillary column, using 99.999% helium as acarrier gas at a constant flow rate (1 mL/ min). The GC column temperature was programmed to hold at 80 ℃ for 30 s and rise to 175 ℃ at a rate of 70℃ per minute, then rise to 230 ℃ at a rate of 8 ℃ per minute, finally hold at temperature of 230 ℃ for 2 min. The injection volume of samples was 1 μL and introduced in splitting mode (50:1) with the inlet temperature of 230 ℃. The ion sources temperature was 230 ℃ and the quadrupole temperature temperature was 150℃. The scanning mode was selected as ion Monitor. Analytes in smaples were identified and quantified by software of Masshunter (v10.0.707.0, Agilent, USA). To draw a linear regression standard curve for sample concentration calculation, the mass spectrum peak area of the analyte was used as the ordinate and the concentration of the analyte as the abscissa.

2.Result

Figure S1

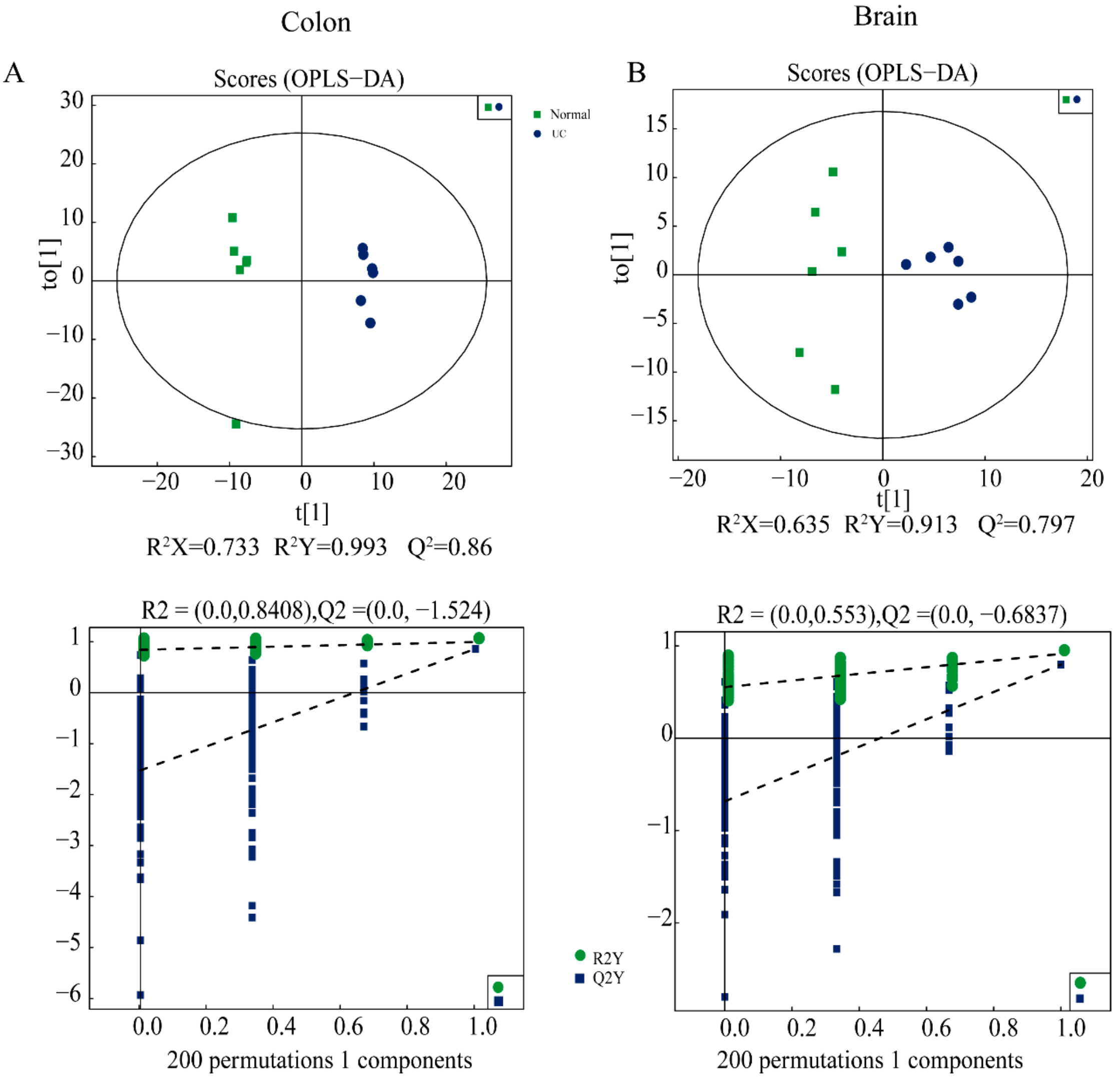

Figure S1.Colon and brain metabolomic analyses between Normal and UC group. (A–B) OPLS-DA model and permutation test of Colon in the two groups. (A) Colon. (B) Brain.

Figure S2

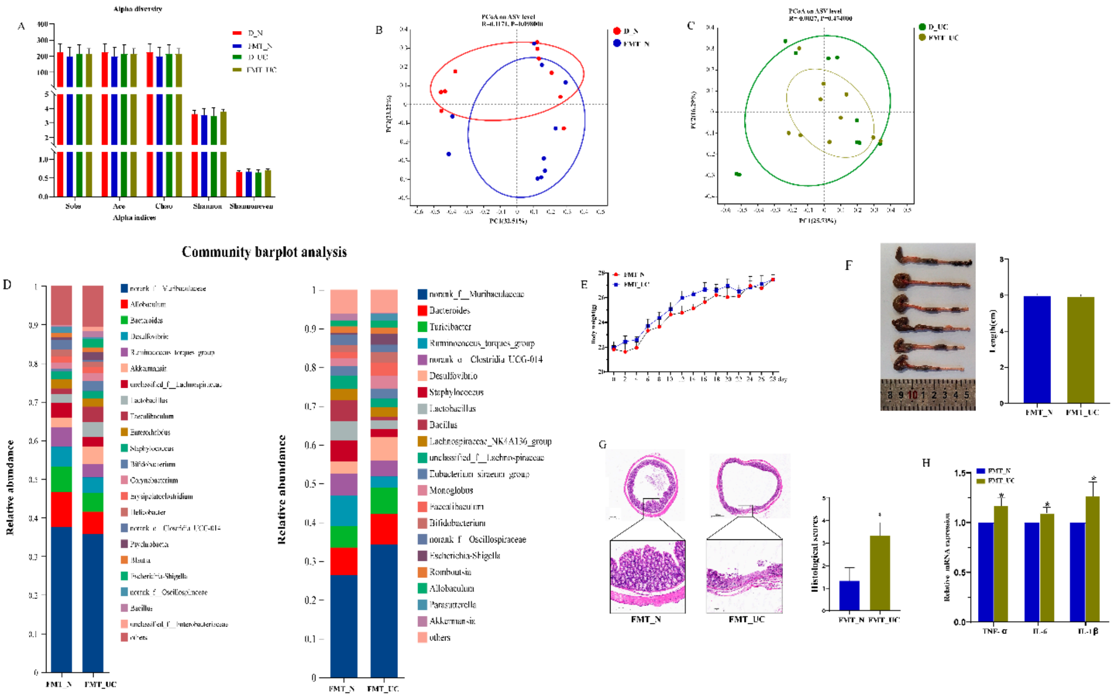

Figure S2. The 16sRNA sequence and colitis change after transplating the feces. (A)Indices of α-diversity measured by *Sobs, Ace, Chao, Shannon,and Shannoneven* in the D_N,FMT_N,D_UC and FMT_UC groups; (B-C)PCoA of the gut microbiome composition on ASV level based on the Bray-Curtis distance for D_N and FMT_N ;D_UC and FMT_UC.Each point indicates samples from mice of each group, which is indicated by a different color and shape. PC: principal component; (D) Bar plot analysis of gut microbiota relative abundance of bacterial genus in D_N and FMT_N;D_UC and FMT_UC.Different colors represent different genus(n=10); (E)The weight chang between FMT_N and FMT_UC group(n=6); (F)The colon length between FMT_N and FMT_UC group(n=3); (G)The HE and histological scores between FMT_N and FMT_UC group(n=3); (H) The expression of IL-6,IL-1β, and TNF-α between FMT_N and FMT_UC group(n=3). Data are presented as the mean ± SD. $*p < 0.05$, $**p < 0.01$. Alpha diversity indices (Sobs, Ace, Chao, Shannon, and Shannon evenness indices) were compared using the Wilcoxon rank-sum test. Principal coordinate analysis (PCoA) based on Bray–Curtis similarities and ANOSIM

analysis were conducted to assess β-diversity. Data are presented as mean ± SD, P values were determined using Student's t-test, *$P < 0.05$, **$P < 0.01$, ***$P < 0.001$.

Figure S3

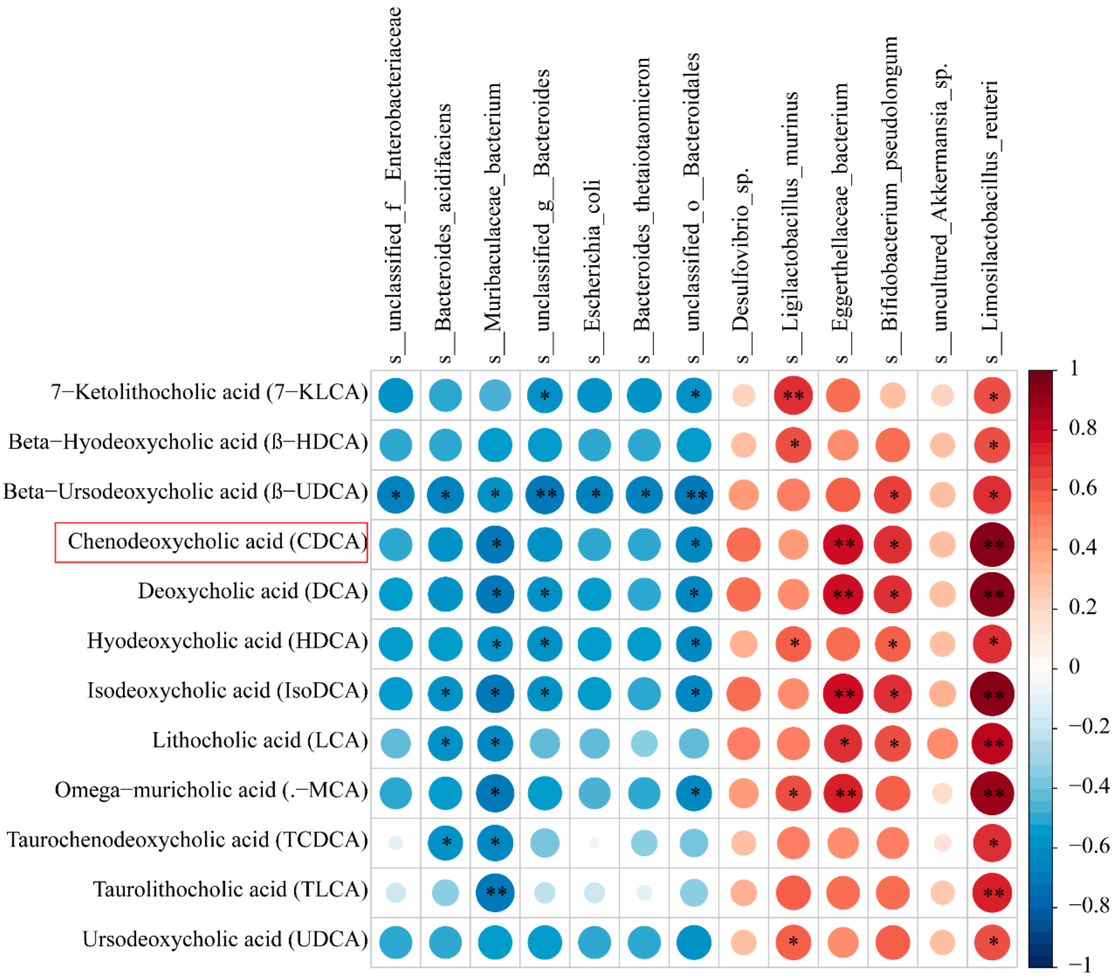

Figure S3. The Heatmap of correlation analysis between different bile acid metabolites in the colon and significant different intestinal bacteria (top 20) (Pearson, *$p < 0.05$, **$p < 0.01$).

Table S1. Significantly different colon and brain metabolites between the Normal and UC groups.

	Colon			Brain		
**Metabolites**	**Normal (Mean±SD)**	**UC(Mean±SD)**	**FC**	**Normal (Mean±SD)**	**UC (Mean±SD)**	**FC**
**Lithocholic acid （LCA）**	3504.23±1394.76	676.08±856.37	0.193	117.18±7.63	80.59±19.38	0.688
**Isodeoxycholic acid (IsoDCA)**	46899.4±22892.71	3377.26±4702.37	0.072	62.06±15.5	38.86±12.12	0.626
**Chenodeoxycholic acid （CDCA）**	125983.24±64803.65	9378.56±12599.34	0.074	141.57±29.58	61.14±5.41	0.432
**Deoxycholic acid (DCA)**	88195.65±43401.33	6420±9045.75	0.073	113.95±26.58	46.15±2.12	0.405
**Glucosamine**	26801.92±9627.75	52394.36±8756.83	1.955	502.41±138.23	1243.06±796.75	2.474
**Sorbitol**	823.58±52.38	567.78±242.36	0.689	422.48±46.8	524.6±77.42	1.242
**Stearylcarnitine**	76706.83±19359.12	30011.47±16397.32	0.391	9183.72±1363.65	5291.33±1385.25	0.576
**Heneicosanoic acid**	52886.26±10046.97	42596.62±3346.8	0.805	47375.1±1741.32	43928.78±3035.14	0.927
**10Z-Nonadecaenoic acid**	9777.81±2623.79	6438.69±837.69	0.658	1616.56±249.3	1193.48±331.57	0.738
**Ricinoleic acid**	146.69±76.43	33.93±11.31	0.231	9.19±1.5	6.49±1.15	0.706
**Anserine**	2788.02±935.87	4578.54±1315.93	1.642	4890.62±553.05	6371.53±635.93	1.303
**Conjugated linoleic acids (CLA)**	331650.62±81714.12	206613.81±77004.29	0.623	21871.98±1961.99	30518.29±3370.11	1.395
**Linoelaidic acid**	277235.32±79134.58	172132.59±63732.73	0.621	15736.65±981.31	21453.85±2294.94	1.363
**Linoleic acid**	228860.46±60958	144911.17±51995.27	0.633	14318.4±1081.46	18577.22±2040.18	1.297
**Gamma-Linolenic acid**	17588.32±8078.76	8428.37±5688.18	0.479	623.27±73.13	791.87±39.47	1.271
**O-Phosphoethanolamine**	2359079.17±1012883.42	1108887.38±881585.28	0.470	5324500.01±621652.23	6539361.35±499439.01	1.228
**Uric acid**	118389.19±29218.32	179204.1±46136.85	1.514	3065.48±536.15	5670.65±1901.36	1.850
**Xanthine**	51420.77±5252.82	25697.39±26863.54	0.500	18149.82±1962.64	23734.09±2128.18	1.308
**Hypoxanthine**	124703.66±22154.79	43678.56±67615.62	0.350	205259.32±12578.29	222570.65±11894.74	1.084
**5-Hydroxy-tryptophan**	2141.01±427.94	3227.52±1090.03	1.507	1410.65±201.99	1782.62±193.03	1.264
**5'-Deoxyadenosine**	26.71±11.61	44.75±4.03	1.676	51.22±9.05	39.82±5.21	0.778

Abbreviations: FC: fold change, unit: *ng/g*

Table S2. The brain protein with the same change trends on the protein and transcriptional levels between Normal and UC groups.

Gene name	FC(gene)	FC(protein)	Gene name	FC(gene)	FC(protein)
Wipf3	1.824263256	2.265	Plin4	14.5131	2.437
Ly6c1	1.376153015	1.532	Il16	1.853263	5.374
Saa1	11.77324053	47.8	Hmgcs1	0.634353	0.6797
Nt5dc2	2.387936206	47.8	Slc5a3	0.706527	0.7305
Frat1	1.259322528	4.489	Serpinh1	0.742106	0.6795
Zbtb16	1.992129159	1.737	Nnat	0.74251	0.1258
Cyp2d22	1.582983638	1.21	Tspan6	0.815277	0.6199
S100a8	11.49515239	9.569	Slc22a8	0.71127	0.5165
Ezr	1.402982931	1.255	Fdps	0.776158	0.7839
Sun2	1.276902037	1.221	Gjb1	0.647566	0.3985
Eml2	1.268122096	47.8	Gap43	0.823151	0.703
Chil3	5.950403403	6.432	Scd1	0.7571	0.3248
Gjb6	1.518592891	3.481	P2ry12	0.628991	0.7771
Slc45a1	1.241186932	47.8	Dgkb	0.753565	0.6661
Shd	1.252992863	1.331	Lpcat2	0.793301	0.6895
Usp54	1.338764507	1.286	Msmo1	0.624549	0.7299
Lrrc1	1.202428178	1.523	Pycard	0.706906	0.8027
Mt2	1.56845384	1.844	Radil	0.751501	0.651
Rbm3	1.81218334	1.351	Fam114a1	0.798501	0.3302
Sult1a1	4.239366849	2.751	Alpl	0.631455	0.7584
Add2	1.219853986	1.328	Ak4	0.822999	0.8001
Lias	1.221729052	1.223	Tmeff1	0.814886	0.8218
Tbc1d1	1.219695863	1.252	Depdc7	0.615019	0.5837
Map3k6	2.25612511	47.8	Plxdc2	0.694542	0.8004
Snrpb	1.24850583	1.621	Fam107b	0.824477	0.8105
Lcn2	20.82804953	47.8	D16Ertd472e	0.741772	0.09603
Serpinb8	1.331387255	1.44	Ncam2	0.720689	0.8158
Bok	1.374020711	1.24	Riox2	0.79182	0.3701
Prelid3a	1.29523593	1.202	Top2a	0.483708	0.4263
Sh3bp1	1.25940756	1.685	Efemp1	0.762341	0.6034
Pdzd2	1.571460029	1.379	Fabp7	0.350607	0.6305
Anxa11	1.470347147	1.872	Smurf2	0.832687	0.352
Fam107a	1.737040993	1.551	Pltp	0.714969	0.7268
Camkk1	1.639653032	1.258	Cacybp	0.771668	0.812
Sf3a2	1.270343575	1.553	Nav1	0.78316	0.7713
Sdc4	1.538102741	1.211	Rcn1	0.76221	0.6375
Htra1	1.449779366	1.602	Shh	0.780544	0.6889
Prodh	1.762316352	1.269			

Abbreviations: FC: fold change
